# Allergic respiratory disease (ARD), setting forth the basics: proposals of an expert consensus report

**DOI:** 10.1186/s13601-017-0150-2

**Published:** 2017-05-18

**Authors:** Ana M. Navarro, Julio Delgado, Rosa M. Muñoz-Cano, M. Teresa Dordal, Antonio Valero, Santiago Quirce, Behalf of the ARD Study Group

**Affiliations:** 1 UGC of Allergy, Hospital El Tomillar , Carretera Alcalá - Dos Hermanas km 6, 41700 Dos Hermanas, Seville Spain; 2 UGC of Allergy, Hospital Universitario Virgen Macarena, Seville, Spain; 3 Allergy Unit, Pneumology Department, Hospital Clinic, Institut d’Investigacions Biomèdiques August Pi Sunyer (IDIBAPS), Barcelona, Spain; 4 Allergy Service, Hospital Municipal, Badalona Serveis Assistencials, Badalona, Spain; 5 Allergy Service, Sant Pere Claver Fundació Sanitària, Barcelona, Spain; 6 Department of Allergy, Hospital La Paz Institute for Health Research (IdiPAZ), Madrid, Spain

**Keywords:** Consensus, Delphi method, Allergic respiratory disease, One airway, Aeroallergens, Allergic asthma, Allergic rhinitis, Allergic rhinoconjunctivitis, Allergen immunotherapy

## Abstract

**Background:**

The variability of symptoms observed in patients with respiratory allergy often hampers classification based on the criteria proposed in guidelines on rhinitis and asthma.

**Objectives:**

We assessed specific aspects of allergic respiratory disease (ARD) that are not explicitly addressed in the guidelines in order to issue specific recommendations and thus optimize clinical practice.

**Methods:**

Using the Delphi technique, 40 Spanish allergists were surveyed to reach consensus on 71 items related to ARD.

**Results:**

Consensus was achieved for 95.7% of the items. These included the following: the clinical manifestations of ARD are heterogeneous and individual airborne allergens can be related to specific clinical profiles; the optimal approach in patients with ARD is based on the global assessment of rhinoconjunctivitis and asthma; aeroallergens are largely responsible for the clinical features and severity of the disease; and clinical expression is associated with the period of environmental exposure to the allergen. Pharmacological treatment of ARD is often based on the intensity of symptoms recorded during previous allergen exposures and cannot always be administered following a step-up approach, as recommended in clinical practice guidelines. Allergen immunotherapy (AIT) is the only option for overall treatment of respiratory symptoms using an etiological approach. AIT can modify the prognosis of ARD and should therefore be considered a valuable first-line treatment.

**Conclusions:**

The present study highlights gaps in current asthma and rhinitis guidelines and addresses specific aspects of ARD, such as global assessment of both asthma and rhinitis or the specific role of variable allergen exposure in the clinical expression of the disease.

## Background

Since the publication of the ARIA document in 2001 [1], the “one airway” concept has been accepted almost unanimously by the medical community to describe specific aspects of patients diagnosed with rhinoconjunctivitis with or without asthma. This concept reflects the obvious epidemiological, pathophysiological, diagnostic, and therapeutic relationship between both disorders. In fact, rhinoconjunctivitis and asthma are considered different manifestations of the same disease, and this observation determines clinical management.

It is therefore surprising that consensus guidelines do not usually consider asthma and rhinoconjunctivitis as one disease that should be managed using a comprehensive approach. Furthermore, the focus of current guidelines is mostly on the pathophysiological, clinical, and therapeutic aspects of rhinoconjunctivitis and asthma, with no emphasis on the etiological factors [2–10]. Nevertheless, allergens play a decisive role in the onset of symptoms and influence the clinical manifestations and progress of both rhinoconjunctivitis and allergic asthma. Current classifications of asthma and/or allergic rhinitis by consensus guidelines cannot be universally applied to patients with allergic respiratory disease owing to their high heterogeneity. Therefore, a comprehensive understanding of patients with allergic respiratory disease (ARD) requires that specific aspects of the etiological agent be addressed in the guidelines.

The present consensus defines the characteristics of ARD and reflects on the peculiarities of the disease as a single entity. This document is based on available evidence and the experience of clinical experts. It provides advice to professionals treating patients whose peculiarities are not explicitly included in guidelines and makes a series of recommendations to address this unmet need.

## Methods

A scientific committee formed by the authors of this manuscript reviewed the relevant medical literature and developed a structured questionnaire to include specific aspects of ARD from routine practice that are poorly covered by current guidelines. Using a modified Delphi methodology [11], 40 expert allergists who were members of the Committees of Asthma and rhinoconjunctivitis of the Spanish Society of Allergy and Clinical Immunology (SEAIC) between 2010 and 2014 (see “Acknowledgements” section) anonymously assessed the 71 statements in 2 consecutive rounds between September and December 2014. The 71 items were divided into 4 blocks as follows: (1) Definition and Epidemiology, (2) Physiopathology and Etiology, (3) Symptoms, Classification, and Diagnosis; and (4) Treatment: Avoidance, Drug Treatment, and Allergen Immunotherapy (AIT).

After analyzing the results of the first round, one of the facilitators provided an anonymous summary of the results, as well as the reasons allergists provided for their judgements. Thus, allergists were encouraged to revise their earlier answers in light of the replies of other members of the panel, and a second round was held to address the remaining questions. A 9-point, single, ordinal, Likert-type scale was used to grade opinion on each item. Following the Delphi categorization, responses were classified into 3 groups: “disagreement” (1–3), “neither agreement nor disagreement” (4–6), and “agreement” (7–9). The survey also offered the possibility of adding individual explanatory observations for each answer. Once the second round was finished, the results were analyzed. The median position of the scores and the level of agreement or disagreement [12] achieved were measured according to the following criterion: consensus was considered to have been reached for an item when no more than a third of the scores were outside the region of three points (1–3, 4–6, 7–9) from where the median was located. In this case, the value of the median score determined the group consensus reached, as follows: “agreement”, majority with medians ≥7; “disagreement”, majority with medians ≤3; “no consensus”, items with medians in the region 4–6 and when the scores of a third or more of the participants were in the region 1–3, and another third or more in the region 7–9. The items for which dispersion of opinions was high (interquartile range ≥4 points) were also considered for assessment.

## Results

In the literature review carried out, we found that most guidelines and position papers on rhinitis [2–6] emphasize the relationship between asthma and rhinitis (Table 1), and specific sections of some asthma guidelines discuss the relationship between asthma and rhinitis [7–10] (Table 2). However, no guidelines consider both asthma and rhinoconjunctivitis as one disease and offer a comprehensive approach.

**Table 1:** Asthma in guidelines on rhinitis

Guideline	Author, year	Chapter	Diagnostic or therapeutic considerations
Clinical practice guideline: allergic rhinitis [6]	Seidman, 2015	Statement 5. Chronic Conditions and Comorbidities: Clinicians should assess patients with a clinical diagnosis of allergic rhinitis for, and document in the medical record, the presence of associated conditions such as asthma, atopic dermatitis, sleep-disordered breathing, conjunctivitis, rhinosinusitis, and otitis media	Evaluation of allergic rhinitis must always include the assessment of asthma. The clinician should inquire about typical symptoms such as dyspnea, cough, wheezing, and exercise-related symptoms. A physical examination should be performed, and the evaluation must be repeated at the follow-up visits, particularly in children. Spirometry must be performed whenever asthma is suspected
Allergic Rhinitis and its Impact on Asthma (ARIA) guidelines: 2010 Revision [5]	Brozek, 2010	VI. Treatment of allergic rhinitis and asthma in the same patient	Recommendations about medical treatment and immunotherapy: subcutaneous immunotherapy (SCIT) and sublingual immunotherapy (SLIT)
The diagnosis and management of rhinitis. An updated practice parameter [4]	Wallace, 2008	Major comorbid conditions Asthma	Lung function tests must be considered in patients with rhinitis Treatment of allergic rhinitis may improve asthma control in patients with coexisting allergic rhinitis and asthma Treatment of allergic rhinitis with intranasal corticosteroids and certain second-generation antihistamines may improve asthma control when both diseases coexist Allergen immunotherapy may prevent the development of new allergen sensitizations and reduce the risk for the future development of asthma in patients with allergic rhinitis
BSACI (British Society for Allergy and Clinical Immunology) guidelines for the management of allergic and non-allergic rhinitis [3]	Scadding, 2008	Co-morbid association Rhinitis and asthma–the link	Treatment of rhinitis is associated with improvement of asthma (Grade of recommendation, A) Patients with comorbid asthma and rhinitis receiving treatment for allergic rhinitis have a significantly lower risk of hospitalization or emergency department visits for asthma
Allergic Rhinitis and its Impact on Asthma (ARIA) 2008 Update [2]	Bousquet, 2008	9. Link between rhinitis and asthma	Allergic rhinitis should be considered a risk factor for asthma along with other known risk factors Patients with persistent allergic rhinitis must be evaluated for asthma based on symptoms, physical examination, and, if possible lung function tests (spirometry pre- and post-bronchodilator). Patients with asthma must be appropriately evaluated (history and physical examination) for rhinitis A combined strategy for the treatment of both upper and lower airway diseases is strongly recommended

**Table 2:** Rhinitis in asthma: guidelines

Guideline	Author, year	Chapter	Diagnostic or therapeutic considerations
GEMA 4.0 [7], Spanish Guideline on the Management of Asthma	Executive Committee of the GEMA, 2015	6. Rhinitis and nasal polyposis	Treatment of rhinitis is indicated in the treatment of asthma Inter-relationships between treatments (anti-leukotrienes, intranasal corticosteroids, immunotherapy) and epidemiological aspects are addressed
GINA 2016, Global Strategy for Asthma Management and Prevention [8]	2016 GINA Report	Part D. Managing asthma with comorbidities and in special populations Rhinitis, sinusitis and nasal polyps	Refers to ARIA
British guideline on the Management of Asthma [9]	British Thoracic Society, 2014	No	Studies confirm that atopic dermatitis and atopic rhinitis are amongst the factors most strongly associated with asthma persisting into teenage years
NAEPP [10], National Asthma Education and Prevention Program	Expert Panel Report 3, 2007	Section 3, Component 3: Control of Environmental Factors and Comorbid Conditions That Affect Asthma Comorbid conditions Rhinitis/sinusitis	It is important for clinicians to appreciate the association between upper and lower airway conditions and the part this association plays in asthma management

With respect to the issues addressed in this study, consensus was achieved for 95.7% (68/71) of the items (agreement, 67; disagreement, 1) (Tables 3, 4, 5, 6). In the first round, consensus was achieved in all but 7. Among the items for which consensus was achieved, it is especially interesting that experts consider that individual aeroallergens may be related to specific clinical profiles and should be taken into account for patient management. In addition, pharmacological treatment of ARD in routine practice is often based on the intensity of symptoms during previous exposures and may not always be established using a step-up approach, as recommended by clinical practice guidelines. As for AIT, the experts think that this approach can modify the prognosis of ARD and should therefore be considered a valuable first-line treatment. No agreement was reached for item 46 (“Patients with ARD sensitized to pollens present symptoms only during the pollen season”).

**Table 3:** Items included in the questionnaire and results

		Mean	Median	Interquartile range	Above the median	Result
1	There is abundant evidence confirming the notion of *one airway, one disease*, which is the conceptual basis of the management of patients diagnosed with rhinoconjunctivitis and/or asthma	8.13	8	1	10	Agreement
2	The definition of allergic respiratory disease (ARD) as a single entity that includes rhinoconjunctivitis and asthma would facilitate its management	7.38	8	2.5	25	Agreement
3	ARD is an altered state of health caused by the generation of IgE antibodies to airborne allergens leading to various clinical manifestations in the upper and/or lower airway	7.85	8	2	10	Agreement
4	The ARD endotype is characterized by the presence of allergic airway inflammation that constitutes the etiological basis of the disease and its exacerbations	7.98	8.5	1.5	12.5	Agreement
5	The clinical manifestations of ARD include nasal (or naso-ocular) symptoms and/or bronchial symptoms	8.55	9	1	0	Agreement
6	The clinical manifestations of ARD may be present perennially or seasonally	8.08	9	1	15	Agreement
7	The clinical manifestations of ARD may be present intermittently or persistently	8.3	9	1	7.5	Agreement
8	The clinical manifestations of ARD may be variable at different times in the patient’s life	8.55	9	1	0	Agreement
9	A comprehensive approach to rhinoconjunctivitis and allergic asthma includes the assessment of both entities, irrespective of whether they are present at a given time in a patient	8.15	9	1	7.5	Agreement
10	The prevalence of ARD depends on the age of the patient	7.93	8	2	7.5	Agreement
11	The prevalence of ARD depends on the clinical manifestations analyzed (rhinoconjunctivitis, asthma, or both)	7.6	8	2	12.5	Agreement
12	The prevalence of ARD has geographic variability.	7.43	8	2	20	Agreement
13	Allergic rhinitis usually precedes the development of asthma in adults	7.7	8	1	7.5	Agreement
14	The probability of developing symptoms affecting the lower airway is increased by up to 3-5 times in patients with ARD expressed as persistent allergic rhinitis	7.83	8	1.5	5	Agreement
15	Rhinoconjunctivitis and asthma may appear consecutively or simultaneously in ARD patients	8.3	8	1	0	Agreement
16	An early assessment of ARD should be in made children with food allergy and/or atopic dermatitis	8	8	1	7.5	Agreement

Definition and Epidemiology

**Table 4:** Items included in the questionnaire and results

		Mean	Median	Interquartile range	Above the median	Result
17	ARD is characterized as an inflammatory process with a characteristic Th2-mediated response profile	8.08	8	1.5	5	Agreement
18	ARD is characterized by inflammation of both the upper and the lower respiratory tract, which may be of different intensity	8.35	8.5	1	0	Agreement
19	Bronchial hyperresponsiveness is observed in more than one-third of ARD patients who have clinical manifestations in the upper airway	8.13	8	1	2.5	Agreement
20	Although no single mechanism fully explains rhinitis-asthma inter-relationships, systemic spread of allergic inflammatory mediators is the most widely accepted pathway	6.55	7	1	30	Agreement
21	Functional impairment of the bronchial epithelium leads to increased susceptibility to infections and facilitates new allergic sensitizations in ARD patients	7.45	8	2	17.5	Agreement
22	The underlying pathophysiological changes are present all year long in ARD patients with only seasonal clinical manifestations, as a result of infections or exposure to environmental irritants	7.5	8	1	20	Agreement
23	Respiratory infections are usually more severe and last longer in ARD patients	7.05	8	1	22.5	Agreement
24	Clinical manifestations are determined mainly by environmental factors but also by genetic factors	6.7	7	3	30	Agreement
25	The presence and persistence of allergens account for the characteristics of clinical manifestations in ARD patients	7.15	7	1	22.5	Agreement
26	Allergen characteristics and type of exposure can partially determine whether rhinoconjunctivitis precedes asthma or both entities develop simultaneously	7.1	7	1	17.5	Agreement
27	Some allergens induce symptoms more frequently in the upper airway than in the lower airway	7.5	8	2	17.5	Agreement
28	Some airborne allergens are related to more severe forms of asthma	7.98	8.5	2	12.5	Agreement
29	In ARD patients, some allergens can cause worse quality of life than others owing to the characteristics of their exposure	7.85	8	2	10	Agreement

Pathophysiology and Etiology

**Table 5:** Items included in the questionnaire and results

		Mean	Median	Interquartile range	Above the median	Result
30	Ocular itching and sneezing (upper respiratory tract) and recurrent wheezing (lower respiratory tract) are the symptoms that best correlate with the diagnosis of ARD	7.33	7	2	25	Agreement
31	The presence of asthma must be evaluated in all patients with allergic rhinoconjunctivitis	8.58	9	1	2.5	Agreement
32	A patient with ARD can manifest allergic rhinoconjunctivitis after being exposed to a specific allergen and asthma after exposure to a different one	7.88	8	1.5	12.5	Agreement
33	In the same patient, the presence of rhinoconjunctivitis and/or asthma at a particular time may depend on the intensity and duration of exposure to the allergen	8.2	8	1	0	Agreement
34	We define the concept of “maximum severity” as the highest intensity of symptoms achieved in previous allergen exposures	7.4	7.5	1	17.5	Agreement
35	Due to the variability of symptoms in ARD patients, it is important to record the “most severe” episodes as well as the symptom-free periods	7.98	8	1.5	10	Agreement
36	The variability of symptoms in ARD patients hampers their classification using the criteria proposed by consensus guidelines	7.85	8	2	12.5	Agreement
37	The current classification used by guidelines is based on the assessment of the intensity and frequency of symptoms of rhinoconjunctivitis and asthma separately and does not assess specific aspects of the causative allergens	8.18	8	1	10	Agreement
38	Besides the intensity and duration, the description of ARD symptoms should consider other aspects such as the frequency of the episodes, seasonality, and recurrence of symptoms at specific times	8.35	8.5	1	0	Agreement
39	A specific classification emphasizing the role of the causative allergen is required for patients with ARD	7.55	8	2	12.5	Agreement
40	A classification considering severity, control level, and clinical characteristics of the airborne allergens is required for diagnosis of ARD and treatment	7.63	8	2	12.5	Agreement
41	Control of ARD varies significantly depending on the intensity of the exposure to the responsible allergen	8.08	8	1	5	Agreement
42	ARD must be suspected on the basis of a compatible history and allergy workup	8.43	9	1	2.5	Agreement
43	Diagnosis of ARD is based on compatible clinical manifestations, the allergological study, and environmental exposure	8.35	9	1	2.5	Agreement
44	An allergological study must be indicated when symptoms of ARD have an impact on a patient’s quality of life	7.03	8	2	22.5	Agreement
45	Precise information regarding the characteristics of a pollen seasons is required for a proper diagnosis	8.23	8	1	5	Agreement
46	Patients with ARD sensitized to pollens present symptoms only during the pollen season	3.08	3	1	17.5	Disagreement
47	Patients with ARD may not meet functional and inflammatory criteria for rhinitis and/or asthma when allergen exposure is not present	7.93	8	2	2.5	Agreement
48	The diagnosis of ARD with lower respiratory tract involvement can be assumed in patients with allergic rhinoconjunctivitis and symptoms of bronchial asthma (even if asthma has not been confirmed by lung function tests)	5.63	7	4	42.5	No consensus
49	Allergy tests (prick tests, specific IgE, specific challenge) are reliable both in and out of the pollen season	8.55	9	1	0	Agreement
50	Lung function tests may be normal out of the pollen season in patients with upper and lower ARD during the pollen season	7.73	8	2	10	Agreement

Symptoms, Classification, and Diagnosis

**Table 6:** Items included in the questionnaire and results

		Mean	Median	Interquartile range	Above the median	Result
51	Treatment of rhinitis in patients with asthma contributes to the improvement of bronchial symptoms	7.7	8	2	7.5	Agreement
52	Treatment of rhinitis in patients with asthma reduces socio-economic costs	7.93	8	1.5	12.5	Agreement
53	Treatment of rhinitis in patients with asthma improves their quality of life	8.33	8.5	1	2.5	Agreement
54	Allergen avoidance in ARD is the first line of treatment for all patients, regardless of severity	7.8	8	2	12.5	Agreement
55	Maintenance drug therapy must be recommended, at least as long as the patient is exposed to the causative airborne allergen	7.35	8	2	22.5	Agreement
56	Maintenance drug therapy can be extended for as long as is necessary to achieve good control of the disease	8.38	9	1	0	Agreement
57	Adjustment of treatment in ARD patients must consider the “maximum severity reached in previous allergenic exposures”	7.28	8	1	15	Agreement
58	Treatment of patients who experienced severe symptoms in previous allergenic exposures may not follow the step-up strategy recommended by consensus guidelines and can begin with a higher therapeutic step	7.85	8	2	7.5	Agreement
59	The doses used in the pharmacological treatment of ARD patients may be greater than those commonly used in non-allergic patients	6.23	7	3	42.5	No consensus
60	The prognosis of ARD depends on the presence of polysensitization	6.6	7	2	30	Agreement
61	The treatment strategy in polysensitized patients consists of adapting maintenance treatment to the relevant allergen	6.95	7	1	22.5	Agreement
62	Failure of drug therapy is not a prerequisite for AIT in patients with ARD	8.35	9	1	2.5	Agreement
63	AIT is most effective in early stages of ARD	7.95	8	1.5	10	Agreement
64	Most patients will benefit from treatment with AIT to slow disease progression	7.75	8	2	15	Agreement
65	Most patients with ARD will benefit from treatment with AIT to reduce the severity of symptoms and use of medication and to improve quality of life	7.95	8	1.5	7.5	Agreement
66	Unlike pharmacological treatment, AIT improves the prognosis of ARD	8.08	8	1	5	Agreement
67	AIT decreases the occurrence of new sensitizations in ARD patients	6.53	7	3	37.5	No consensus
68	AIT can prevent the development of bronchial symptoms in patients with rhinoconjunctivitis	7.85	8	2	10	Agreement
69	In ARD patients, identification of the airborne allergen that is clinically responsible for symptoms is essential when attempting to establish the indication of AIT	8.7	9	0.5	0	Agreement
70	The composition of immunotherapy in polysensitized ARD patients must be based on a selection of the relevant allergen(s) according to the patient’s clinical and sensitization profile	8.3	9	1	2.5	Agreement
71	A sufficient dose of each allergen must be ensured in AIT with mixtures of allergens in polysensitized ARD patients	8.23	8.5	1	5	Agreement

Treatment–avoidance, drug treatment and allergen immunotherapy (AIT)

*ARD* allergic respiratory disease, *AIT* allergen immunotherapy

Consensus was not reached on 3 items in the diagnosis and treatment blocks, as follows: “The diagnosis of ARD with lower respiratory tract involvement can be assumed in patients with allergic rhinoconjunctivitis and symptoms of bronchial asthma (even if asthma has not been confirmed by lung function tests)” (item 48); “The doses used in the pharmacological treatment of ARD patients may be greater than those commonly used in non-allergic patients” (item 59); and “AIT decreases the occurrence of new sensitizations in ARD patients” (item 67).

Detailed results for each item (mean, median, percentage of distribution of respondents located outside the region of the median, interquartile range, and consensus result) are shown in Tables 3, 4, 5 and 6.

## Discussion

The “one airway, one disease” concept [13] has successfully taken root in the medical community, although it is far from being a reality in clinical practice. In fact, there are currently no consensus guidelines for ARD patients. Thus, management is not based on homogeneous criteria and requires the use of 2 separate guidelines, 1 for asthma and 1 for rhinoconjunctivitis.

This consensus study aimed to collect expert opinions from Spanish allergologists about the symptoms, classification, diagnosis, and treatment of ARD to provide a comprehensive approach for clinical practice. A major goal was to address the importance of the allergen as the modulator of individual variability in clinical expression based on the duration and intensity of exposure.

### ARD: Definition

Given the publication of the ARIA guidelines in 2001 [1], the panel agreed that “there is abundant evidence confirming the notion of *one airway, one disease* as the conceptual basis of the management of patients diagnosed with rhinoconjunctivitis and/or asthma” (item 1). Therefore, it follows that “the definition of ARD as a single entity that includes rhinoconjunctivitis and asthma would facilitate its management” (item 2), especially when allergy is its main cause. Finally, the experts of this consensus agreed on the definition that “ARD is an altered state of health caused by the generation of IgE antibodies to airborne allergens leading to various clinical manifestations in the upper and/or lower airway” (item 3).

Allergic inflammation is present in both the upper airway and the lower airway [14, 15], although it may be of locally different intensity (items 4, 17, 18). Therefore, a unified assessment of the airway is necessary, irrespective of whether symptoms of both asthma and rhinoconjunctivitis are present at a given time in a patient (item 9).

The concept of ARD is based on the allergic origin of the disease, and its clinical spectrum includes conjunctivitis, rhinitis, and/or asthma. Not all clinical manifestations must occur simultaneously in ARD patients, although the risk of developing the other clinical manifestations of ARD in the future is greater than in the general population [16].

### The allergen as a key factor in ARD

In ARD patients, allergens and clinical exacerbations are the main triggers of inflammation (acute and chronic). The ARD consensus highlights the importance of considering allergic sensitization in diagnostic and therapeutic decisions.

Various airborne allergens can induce a variety of respiratory symptoms with a wide spectrum of severity [17]. Furthermore, sensitization to several agents (polysensitization) can also substantially modify the clinical features and prognosis of ARD patients [18]. As shown by several studies, specific allergens more frequently induce symptoms in the upper respiratory tract than in the lower respiratory tract (item 27) [19]. In addition, some airborne allergens are related to the most severe forms of asthma (item 28) [20] or persistent forms of asthma [21], and some allergens can lead to worse quality of life than others owing to the characteristics of their exposure (item 29) [22]. Age at sensitization and allergen involved have even been linked to the appearance of specific symptoms [23]. Sensitization to certain allergens, for instance *Alternaria* species, has also been noted as a risk factor for exacerbations [24], severe exacerbations, and even death from asthma [25]. Furthermore, recent studies have linked specific allergens to various late reactions in asthma: whereas house dust mites induce more severe late reactions than pollens, animal dander allergens are related to reactions of intermediate intensity [26].

Other factors modulate the clinical response to the allergen. These include “allergenic pressure”, which is the combination of both intensity and duration of exposure to an airborne allergen. The experts agreed that “a patient with ARD can manifest allergic rhinoconjunctivitis after being exposed to a specific allergen and asthma after exposure to a different one” (item 32) and “in the same patient, the presence of rhinoconjunctivitis and/or asthma at a particular time may depend on the intensity and duration of exposure to the allergen” (item 33).

For the experts consulted, unlike non-allergic asthma or rhinitis, “control of ARD varies significantly depending on the intensity of exposure to the responsible allergen” (item 41).

Contact with an allergen causes pathophysiological changes that affect the development of symptoms triggered not only by allergens, but also by other agents, such as infectious microorganisms (item 23). These symptoms are more intense when patients are exposed to both an allergen and an infectious agent [27]. Recent studies have linked the persistence of asthma after removing the allergenic trigger in individuals with ARD with the activation of Th2-mediated myeloid dendritic cells [28]. The experts agreed that the allergic nature/substrate of ARD might also influence the persistence of respiratory symptoms during periods of no exposure to an allergen (item 46).

### Specific aspects of the diagnosis of ARD

The expert panel agreed that control of ARD depends on a comprehensive diagnosis, including identification of the causative allergen/s and its/their clinical relevance (item 41).

It is well known that “patients with ARD may not meet functional and inflammatory criteria for rhinitis and/or asthma when allergen exposure is not present” (item 47), as occurs in individuals sensitized to pollens out of season [29].

Allergen exposure can influence the results of the diagnostic tests most commonly used in rhinoconjunctivitis and asthma. Whereas allergy tests (skin prick test, specific IgE, allergen challenge) are still useful when patients have no symptoms (item 49), lung function tests may fail to detect bronchial involvement (item 50). Thus, the diagnosis of allergic rhinitis can be made independently of the allergenic exposure. However, according to guidelines, diagnosis of asthma requires the objective demonstration of lower respiratory tract involvement (reversible obstruction, hyperresponsiveness) [7].

### Specific aspects of treatment of ARD: drug therapy

The expert panel agreed that the therapeutic and diagnostic approach to ARD patients cannot be solely and strictly based on the recommendations of current guidelines. Adjustment of drugs and doses is based on the severity of symptoms in previous allergen exposures and does not follow the frequently recommended “step-up” strategy (item 58), especially in patients with seasonal manifestations.

Although a personalized treatment plan is recommended, we may use the “maximum severity of symptoms recorded in previous exposures” as a guide to establishing future treatments (item 57). This must be registered in the medical history (Items 34 and 35) and is particularly important if therapeutic recommendations are given when patients are not exposed to the allergens.

In the opinion of the expert panel, unlike non-allergic rhinitis and asthma, maintenance therapy may only be administered to ARD patients during allergen exposure (item 55) [9]. However, maintenance therapy may also be used over longer periods to ensure good control (item 56).

### Specific aspects of treatment of ARD: AIT

As suggested previously [30], there is a common underlying pathogenic mechanism in all patients with ARD, despite differences in clinical manifestations and types of allergic sensitization. Identification of the causative allergen and prescription of an allergen-oriented treatment improve disease control and prognosis, irrespective of whether asthma and rhinoconjunctivitis appear simultaneously or sequentially. Allergen immunotherapy (AIT) is an etiology-based treatment and should be considered a first-line option in ARD based on the clinical relevance of allergen sensitization, in which exposure to an allergen elicits allergic symptoms with significant intensity or duration.

However, contrary to published evidence [31] and the opinion of the expert panel, some guidelines [8, 9] do not consider AIT to be first-line treatment. The experts agreed that “failure of drug therapy is not a prerequisite for AIT in patients with ARD” (item 62), and that “most of these patients will benefit from treatment with AIT to slow disease progression” (item 64). This consensus advocates for early indication of AIT under the premise that immunotherapy is most effective in the early stages of ARD (item 63) when the optimal dose is applied, thus combining efficacy and safety.

“Unlike pharmacological treatment, AIT improves the prognosis of ARD” (item 66), mostly in monosensitized patients and when an adequate immune response is observed [32]. There is sufficient evidence to support the observation that “most patients with ARD will benefit from treatment with AIT to reduce the severity of symptoms and the use of medication and to improve quality of life” (item 65) [33–35]. Likewise, substantial evidence indicates a preventive effect in the progression from allergic rhinitis to asthma [36] (item 68), especially in children [37].

Some of the authors on the panel agreed that “AIT decreases the occurrence of new sensitizations in ARD patients” (item 67) [38, 39], although consensus was not reached. The experts considered that only some studies in children treated with pollen AIT have demonstrated the development of fewer new sensitizations when compared with those not treated with AIT. Furthermore, this has not been demonstrated for every allergen or in adults treated with AIT.

Polysensitization is an important factor when determining the prognosis of ARD and the indication for AIT (item 70). In polysensitized patients, both maintenance treatment strategies (item 61) and AIT composition (item 69) must be tailored after taking into consideration the most clinically relevant allergen. Therefore, AIT has proven to alleviate patients’ overall symptoms owing to its effect on reducing the most relevant allergen-related symptoms [40].

However, polysensitization does not necessarily mean polyallergy [41]. Molecular diagnosis and knowledge of the predominant allergen are very useful for selecting genuinely polyallergic patients to receive AIT. It has been shown that the final composition of the AIT prescribed may need to be modified in up to 50% of patients when molecular diagnosis is used instead of the classic approach [42].

The inclusion of more than 1 allergen in AIT must be considered when there is more than 1 relevant allergen. The authors of this consensus advocate administration of the complete doses of each allergen to ensure the effectiveness of AIT, although this issue warrants further research (item 71).

### Classification of patients with ARD

ARD is not reflected in the main clinical practice guidelines. Consequently, given that allergy is the most important cause of persistent rhinoconjunctivitis and asthma, the absence of specific references to patients with ARD [9] is remarkable. It is also interesting that the defining characteristics of ARD, such as the clinical variability conditioned by allergen exposure, have not been assessed. Therefore it is difficult to classify ARD patients according to the criteria currently proposed by guidelines (item 36).

The difficulty in fitting patients diagnosed with ARD with the guidelines lies in the fact that “the current classification is based on the assessment of the intensity and frequency of symptoms of rhinoconjunctivitis and asthma separately and does not assess the specific aspects of the causative allergens” (item 37). However, the expert panel agreed that “besides the intensity and duration, the description of ARD symptoms should also consider other aspects such as the frequency of the episodes, seasonality, and recurrence of symptoms at certain times” (item 38). The assessment of these aspects would enable a better approach in ARD patients.

The dynamic nature of allergic diseases has previously been described [37]. Indeed, “the clinical manifestations of ARD may be variable at different times in the patient’s life” (item 8), with variation in the preponderance of nasal over bronchial symptoms [43]. Therefore, appropriate control of these patients requires the evaluation of the whole airway, even though symptoms may not be present at a given time.

**Fig. 1:**
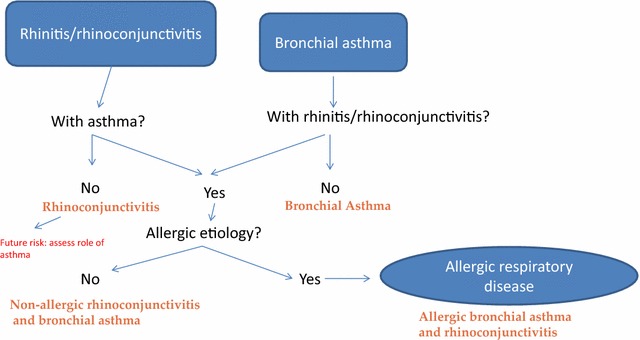
Flow chart for diagnosis of allergic respiratory disease

**Fig. 2:**
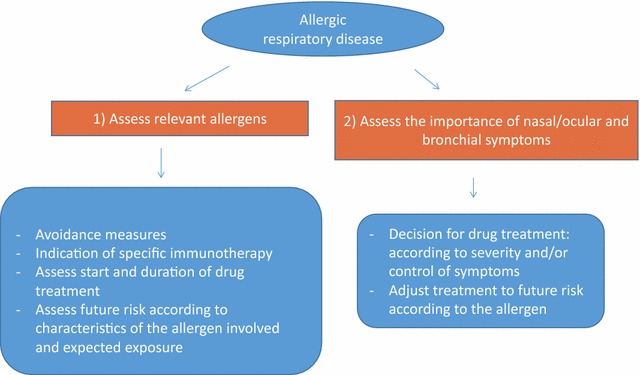
Flow chart for treatment of allergic respiratory disease

The panel of experts highlighted the existence of several unmet needs. 1) Patients diagnosed with ARD require a specific classification that gives prominence to the causative agent (item 39). 2) It is necessary to propose a classification for diagnosis and treatment of ARD that simultaneously takes into account the severity, control, and clinical characteristics of the airborne allergens involved (Item 40). 3) The development of diagnostic and therapeutic approaches that take allergen exposure and the patient’s environment into account would be useful in daily clinical practice. Multiple allergens are frequently implicated in ARD, making it very difficult to identify the most important one. Furthermore, we must bear in mind the existence of other factors not related to the allergen that might contribute to the onset of symptoms. 4) Rhinitis and asthma are currently classified, treated, and evaluated using different guidelines. However, the expert panel recommends a holistic approach to ARD patients, taking into account the clinical expression of respiratory disease at different levels and including its severity and level of control after treatment (Figs. 1, 2). It would be desirable to use questionnaires on disease control [44] and quality of life [45] to provide a global evaluation of ARD.

## Conclusions

Despite the almost unanimous acceptance of the “one airway, one disease” concept, the current consensus guidelines apply two different standards for the management of patients with ARD. As far as we know, no one has previously addressed the need for a global approach to ARD. Therefore, the expert panel proposes a series of recommendations based on the specific aspects of allergic patients with rhinitis and asthma that can be useful in daily clinical practice (Table 7).

**Table 7:** Allergic respiratory disease (ARD): key points

Allergic respiratory disease (ARD) includes patients with clinical manifestations of rhinoconjunctivitis and/or bronchial asthma of allergic etiology
The optimal approach to ARD involves the simultaneous assessment of the upper and lower respiratory tract, irrespective of whether there are symptoms at a given time in a given patient
The clinical features of patients with ARD depend (in part) on the allergen that caused the symptoms and the characteristics of the exposure
The causative allergens of ARD must play a greater role in the choice of treatment
Decisions on drug treatment in patients with ARD may be affected by the clinical severity of previous allergen exposures and not follow the phased strategy suggested by guidelines
Allergen immunotherapy is a comprehensive etiological approach that can modify ARD. Failure of drug therapy is not a prerequisite for allergen immunotherapy in ARD patients

ARD patients are characterized by the presence of allergic rhinoconjunctivitis and/or asthma. The most suitable approach to these patients involves the assessment of all clinical manifestations of the disease, including both rhinoconjunctivitis and asthma, irrespective of whether they are present at a given time.

The clinical manifestations of ARD are variable and related to allergen exposure. Different airborne allergens can be related to specific clinical profiles in patients with ARD. Thus, the causative allergen must play a greater role in decisions on diagnosis and therapy, since the duration and severity of the disease are determined to a large extent by the allergen.

Pharmacological treatment is often chosen based on the severity of symptoms reached in previous allergenic exposures. Treatment with AIT is a comprehensive and etiological approach to the “one airway” disease. Therefore, AIT must be considered a first-line treatment and indicated in the early phases because, unlike pharmacological treatment, it can modify the prognosis of the disease.

### Unmet needs

The peculiarities of ARD are not adequately reflected in the classifications of rhinitis and/or asthma proposed in current guidelines. Therefore, the expert panel considers the development of guidelines that recommend a comprehensive approach to patients with respiratory allergy to be an unmet need.

## Abbreviations

ARD: allergic respiratory disease
AIT: allergen immunotherapy
SEAIC: Spanish Society of Allergology and Clinical Immunology
